# Motion

**Published:** 1874-04

**Authors:** 


### MOTION.

"Music began and glittering stars awoke,
And brilliant suns upon creation broke."

All nature moves. The flight of stars and of luminous comets, that are supposed to shake from their fiery mane pestilence and war, that careers through the illimitable fields of Heaven and then returning homeward, merge their brilliant trains in the dazzling splendor of the sun.

The air, quickening creation with vital life like a human pulse; the perpetual motion of the waters, leaping in cataracts like blood in human arteries and coursing through the deep veins of the world, singing and flashing in the brooks, rippling and rushing in the rivers, and swelling in the billows of the grand old ocean, or glittering in sun-lighted dews, shining in "heaven's bright arch," to remind us of heaven and of God's covenant,—all these are instinct with the greatness, the sublimity of nature in motion.

Nations are restless since Abraham said unto Lot, "if thou will take the left hand, then I will take the right, or if thou depart to the right hand, then I will to the left," there has been a continuous motion of the human family. Upon the ruins of exploded systems splendid empires have arisen like Phœnix from his ashes.

Nation's formularies—sects—have been caught up in the terrible whirlwind of revolution, or borne away in the eddying whirl of empires—hearts beaten into the dust beneath the hoof of the wild war-horse—human bodies have been crushed and mangled, but order and quiet and progress and all the arts of peace have arisen. In the frozen and ice-ribbed Arctic regions, huge mountains of crystal-like ice float about and glow and flash forever in the dim sunlight. Even those terrible icy chains can not bind nature, she rends the Polar icebergs, and sustains her eternal principle of life motion. It is God's life.

With the genesis of the world began an eternal exodus when God by his plastic call brought together the latent elements of an unmade world; when God's voice startled the regions of "night an chaos" where glimmering light shot far into the bosom of darkness, radiaing and flashing; when old Sol, in his glowing and dazzling chariot, entered the fields of heaven, a vital, living God was felt. We hear his chariot in the rustling winds, his voice in the thunder—we feel his breath in the gentle breeze, we see him in the ocean that mirrors the deep blue heaven above, and embraces stars in its folds.

It is not then an unmeaning injunction of the God-head that we shall live by industry. It rests not alone upon man, but upon every living thing that inherited that sad fate. Creeping insects; the fierce tiger and roaring lion; the chirping little bird and the soaring eagle; the little minnow and the huge whale; the slimy snake in the grass and the mighty serpent must all live by motion—industry. The great embodiment of earth's wisdom—Solomon—realized it and pointed to the little ant as an example of industry, engaged noiselessly and sublimely in its mission of preparing an ingenious home, and amply storing it with material to supply its wants. As without motion—as without this vital energy in the physical universe, systems would be involved in a wreck of worlds and crush of matter, so without industry, blight and ruin would accrue to mankind. It is not only true that the earth trembles beneath the mighty vanguard of empire and civilization, moving toward the setting sun, but investigation, &c., are following this progressive march. The oriental traveler—walking amid the ruins of Babylon or Thebes, that once boasted of her hundred gates; or Jerusalem, through whose streets crowding, bustling multitudes once poured—is reminded of the migratory disposition of the human race, and he beholds, in the crimson west of an Italian sky, the light that streams upon the path of man. The smoke of the evening incense no longer ascends to heaven; no more hecatombs are paid; no more the children of Israel, with their young prophet, Daniel, who could read the mysterious hand-writing on the wall, retire to the willow groves and palm trees by the shores of the Euphrates and hang their harps upon the clustering boughs with no heart for joy and song, and lament the cruel persecution inflicted by a riotous king. The ruins that everywhere meet the gaze seem to whisper, *They are gone!* It is all still and solemn. Here, in the path of the great army of Xerxes, is nothing but desolation. The earth, in its diurnal revolution, whirling on its grand career, has sent the tide of emigration rolling westward forever.

Nature teaches by example. How sublime her lessons! We all have, however humble our position may be, a work to perform on this great field of action; and however small may be the work that falls to our share, we should remember that we will be held to a strict accountability for its faithful performance at the bar of the Eternal. However small the work we are called to do, we have no right to avoid it or refuse its performances. Let us, then, strive to emulate the shining and constant example of Nature's Workman (so to speak). Like the little ray of light which pierces the dull earth and warms its cold bosom into life, making it teem with golden harvests, green fields, and with glorious images of beauty; the soft breeze which fans our cheeks, fills our lungs, and murmurs among the trees to breathe its whispered music in our souls; like a kind word spoken in season to point some soul the way to Christ, or to cheer the broken heart and wounded spirit which is, in the eyes of Him who sees the inmost heart, more valuable than the earthly deeds of the mightiest of our race, and will be remembered in heaven when all the civil wreaths and glittering crowns of statesmen, kings, and warriors shall have failed and are lost forever in the depths of oblivion. As a cup of cold water given through proper motives and in the name of Jesus, will be a richer treasure in heaven than the noblest deed that was ever achieved by warrior or statesman, such a deed of charity, small as it may seem, will outshine the stars, and brighter than the combined glories of a thousand suns. Let us, then, press forward with the high resolve of doing our whole duty to God and to society, and though we may never meet in this pathless sea men call life, we will when the beacon light of eternity dawns upon the failing sight, and the heaven of eternal rest opens its golden portals to receive the weary wanderer. Then be of good cheer, my troubled reader: remember that whatever may befall you, your Heavenly Father, who teaches the hearts and souls of His children, will never forsake you if you will listen, and profit by the lessons of divine wisdom which he inculcates. Work! Keep in motion!

> "Let all the ends thou aim'st at
> Be thy God's, thy country's and thy truths."

In the absence of something better, the senior editor wrote the above thoughts just as they hurriedly crowded upon his mind, with the hope that some good may result from them. T. S. P.

